# Effect of root canal rinsing protocol on dentin bond strength of two resin cements using three different method of test

**DOI:** 10.4317/jced.52674

**Published:** 2016-07-01

**Authors:** Maryam Khoroushi, Mohammadreza Sheikhi, Amirhossein Khalilian-Gourtani, Bahram Soleimani

**Affiliations:** 1 DDS, MSc, Professor. Dental Materials Research Center and Department of Operative Dentistry, School of Dentistry, Isfahan University of Medical Sciences, Isfahan, Iran; 2 DDS. Torabinejad Dental Research Center, School of Dentistry, Isfahan University of Medical Sciences, Isfahan, Iran; 3 BS candidate. Electrical engineer, Department of Electrical and Computer Engineering, Isfahan University of Technology, Isfahan, Iran; 4 BS, PhD, Assistant Professor. Department of Health and Statistics, Islamic Azad University, Najafabad, Isfahan

## Abstract

**Background:**

Different studies have used different tests to evaluate bond strength of resin cements to root dentin. In this *in vitro*study, three different tests were used to evaluate the bond strength of two resin cements to root dentin using two root dentin irrigation protocols.

**Material and Methods:**

Ninety-six intact single-rooted teeth were selected for this study. Forty-eight teeth, with a root length of 15mm, were randomly divided into two groups and irrigated with normal saline or 2.5% sodium hypochlorite solutions during root canal preparation, respectively. For each 12 specimens from each group, fiber post #1 was bonded using an etch-and-rinse (Duo-Link) and a self-adhesive (BisCem) resin cement, respectively. After incubation, two specimens were prepared for the push-out test from the middle thirds of the roots. In another 24 teeth, after two 1.5-mm sections were prepared from the middle thirds of the prepared roots, sections of the post were bonded in two subgroups with each of the cements mentioned above and the samples were prepared for the pull-out test. For shear test, the crowns of 48 teeth were cut away, the dentin surfaces were prepared, the two irrigation solutions were used, and the resin cements were bonded. Data collected from the three tests were evaluated by ANOVA, post-hoc Tukey and Weibull tests (α=0.05).

**Results:**

There were significant differences in the mean bond strength values between the three bond strength tests (*P*<0.001). Rinsing protocol and cement type resulted in similar variations in the mean bond strength in all tests (*P*>0.05).

**Conclusions:**

Under the limitations of the present study, the method of the test used had an effect on the recorded bond strength between the resin cement and root dentin. Cement type and irrigation protocol resulted in similar variations with all the tests. Push-out and shear tests exhibited more coherent results.

** Key words:**Bond strength, endodontically treated tooth, fiber post, resin cement, sodium hypochlorite.

## Introduction

Posts are used to provide retention for crown restorations in teeth which have undergone root canal therapy and have lost a large portion of their crown. Two factors are important in selecting a post: strength and esthetic (1). Prefabricated metallic posts and cast posts have been used for many years to achieve retention and strength. However, in recent years non-metallic posts have been marketed in response to ever-increasing demands for esthetic tooth-colored posts, which include epoxy resin posts reinforced with carbon fibers, epoxy resin or dimethacrylate posts reinforced with quartz or glass fibers, zirconia posts, and posts reinforced with polyethylene fibers (2,3).

Use of metallic posts leads to a heterogeneous structure consisting of dentin, the metallic post and the core material, resulting in concentration of stresses on the apical segments of the root, which might increase the risk of vertical root fractures. Other disadvantages of metallic posts include low retention, low esthetic appearance, risk of corrosion, and allergic reactions (1-3). In contrast, the most important advantage of fiber posts is a modulus of elasticity similar to that of dentin, which is almost 20 GPa, resulting in a homogeneous distribution of stresses in tooth structure and its surrounding structures, thereby protecting the root against fracture. Some other advantages of fiber posts over cast posts include esthetic, absence of corrosion risk, lower costs, easier handling, and lack of any need for long and expensive laboratory procedures, and easier preparation with lower risks of endodontic retreatment. The most common failure mode of these posts is deboning at post-resin or resin‒root dentin interface, which does not result in any damages to the main structure of the root in the majority of cases and is considered a favorable failure with the capacity for repair (1,4-8).

It is necessary to create a proper bond at dentin‒resin cement interface and at post‒composite resin core interface for the homogeneous distribution of occlusal stresses and creation of a “monoblock” structure (2,8). Although the most common reason for the failure of restorations retained by posts has been reported to be deboning at dentin‒resin cement interface, the resin ce-ment‒composite resin core interface, too, has an important role in the efficacy and longevity of the restoration (9).

In this context, at present various resin cements are available, which make use of etch-and-rinse and self-etch systems to bond tooth-colored posts to tooth structure (10,11). In addition, during cleaning and preparation of root canals during endodontic treatment various irrigation and disinfecting solutions are used in addition to water (12-14). Sodium hypochlorite is widely used as an irrigation solution in endodontic treatment (13). According to some studies, use of this solution decreases the bond strength of composite resin to dentin (14-18). Generally, use of various cements and adhesives, fiber posts with different chemical compositions, and different solutions for irrigation of dentin have affected the bond strength of fiber posts to root dentin (1,14).

Different studies have used different techniques to evaluate the bond strength of different kinds of posts and resin cements to root dentin, which include shear, push-out, microtensile, pull-out, modified pull-out and modified push-out tests (3,7,19). Each test has its advantages and disadvantages. In the microtensile bond strength test there is the possibility of premature fracture of the samples and the data reported are very diverse. In the push-out test the odds of premature fractures of the samples are low and it is possible to compare the bond strength at different parts of the root because in this test cross-sections of the root and post cemented to it are prepared, with dimensions measured in millimeters or tenths of a millimeter. However, in this test it is difficult to exactly determine the fracture location. On the other hand, retention of air bubbles is possible in this test and the researcher might not be able to make accurate evaluations. Recently, pull-out and modified pull-out tests have been introduced in order to concentrate fracture at a certain interface (7,19,20). In recent years, different tests have been evaluated by researchers and the results have been published. In a recent study an attempt was made to determine to what extent the results of these studies can be compared. According to the results of comparisons made between microtensile, push-out, pull-out and modified push-out tests, designing of samples (including the geometric form and preparation technique) have an effect on the biologic behavior of samples in different bond strength tests (19).

Since factors such as the material type and the bonding interface in samples with different geometric shapes possibly change the results, it might be of interest to evaluate and compare these tests when different materials and protocols are used. In addition, evaluation of the relationship between laboratory tests might help interpret and understand the results of bond strength tests in different studies (7,20). Moreover, previous studies have not determined to what extent the effect of each increasing or decreasing variable would be different in each test type. Therefore, the aim of the present study was to compare the bond strength of two resin cements to root dentin using three commonly used tests, including push-out, shear and modified pull-out tests. The null hypothesis of this study stated that the test type has no effect on the bond strength of an etch-and-rinse and a self-etch resin cement to root dentin prepared with two irrigation protocols.

## Material and Methods

More than one hundred sound anterior maxillary teeth with a root length of >15 mm were selected for the purpose of this *in vitro* study. The teeth were stored in 0.2% thymol solution at 4°C for no more than 3 months after extraction and used for the purpose of the present study after informed patient consent was obtained, based on the guidelines of the Medical Ethics Committee of University of Medical Sciences. After rinsing and 24 hours of storage in distilled water the tooth crowns were removed and all the teeth were radiographed in a manner to reveal the bucco-lingual dimension of each root. Only teeth with one root canal were selected and prepared as follows for push-out, modified pull-out and shear tests.

-Push-out bond strength test 

A total of 24 teeth were filed and flared up to file #60. Half of the samples were irrigated with normal saline (NS) and the remaining half were irrigated with 2.5% sodium hypochlorite (SHC) and then irrigated with NS during root canal treatment. All the canals were shaped and prepared using the fiber post drills. In each group, half of the teeth received #1 silanated fiber post with a diameter of 1 mm, cemented with an etch-and-rinse resin cement (Duo-link, Bisco); the remaining half received the same fiber post which was cemented with a self-etch self-adhesive resin cement (BisCem, Bisco) according to manufacturer’ instructions ( Table 1). After 24 hours of storage under 100% humidity at 37°C, two 1.5-mm sections were prepared from the middle portion of each root using a diamond disk in a cutting machine (Jota, 509348, Germany). The prepared sections underwent a compressive force from the smaller cross-section toward the bigger cross-section at a crosshead speed of 1 mm/min until bond failure in a universal testing machine (Walter & Bai, K21046, Lohningen, Switzerland). The force used was recorded and data was analyzed using SPSS 16 statistical software. *p*-value of <0.05 was set to be statistically significant.

**Table 1:**
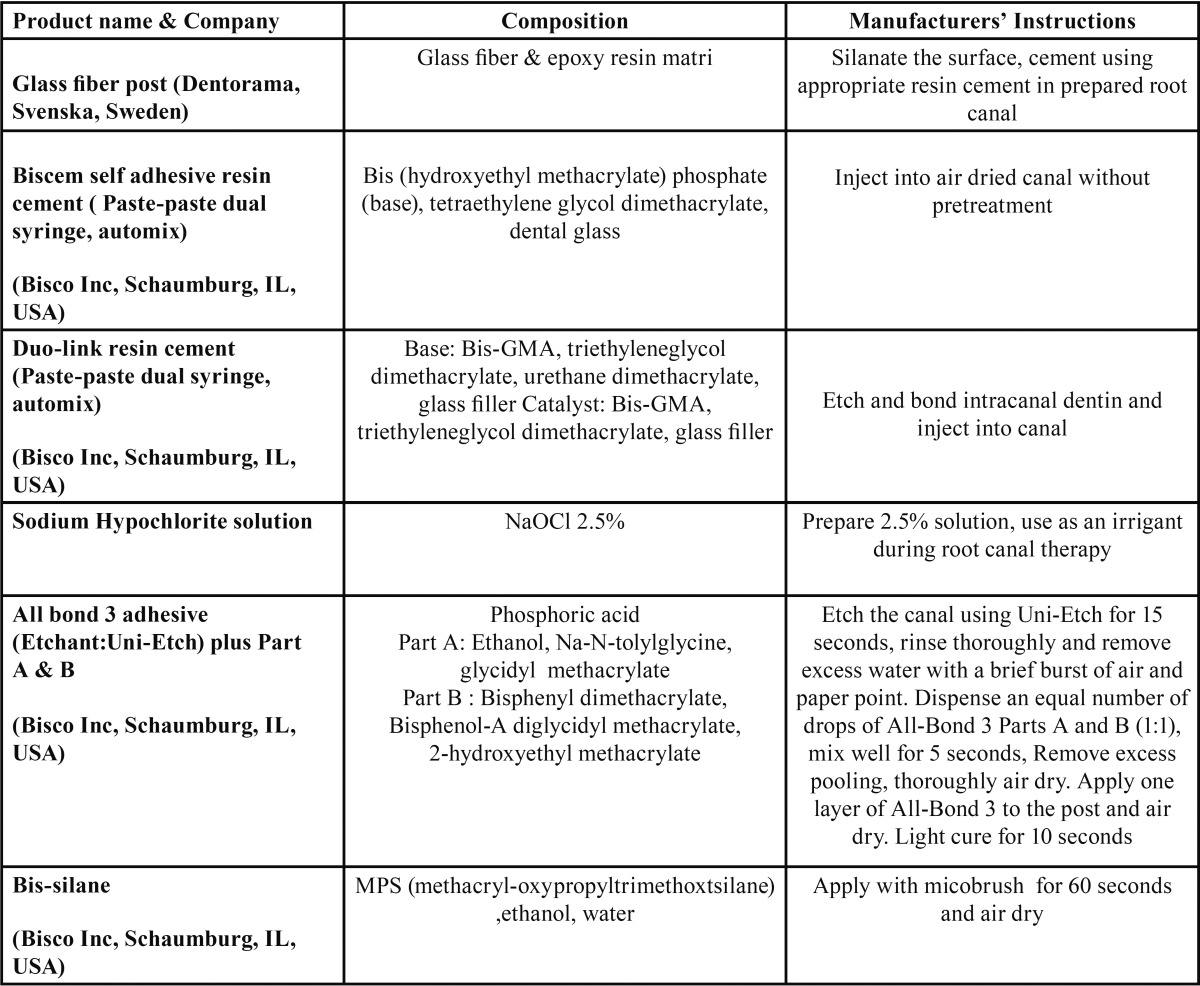
Materials used in the study and mode of their applications according to the manufacturers’ instructions.

-Modified pull-out bond strength test

For the pull-out groups, each 1.5-mm dentin slice was luted with a 4-mm fiber post piece. For this reason, a total of 24 teeth were prepared in a manner similar to that explained for the push-out test. Half of the teeth were irrigated with NS and the other half with 2.5% SHC, followed by NS, during root canal preparation. Two 1.5-mm sections were prepared from the middle portion of each root. The sectioned posts, measuring 4 mm in length, were silanated and cemented using Duo-link and BisCem resin cements. After 24 hours of storage under 100% humidity and mounting in acrylic resin, the posts underwent a pull-out force at a crosshead speed of 1 mm/min using a universal testing machine (Walter & Bai, K21046, Lohningen, Switzerland). The force was recorded and data were analyzed and compared using SPSS statistical software. *p*-value of <0.05 was set to be statistically significant.

-Calculation and equalization of bonded areas for the shear test based on two other tests

**Figure 1:**
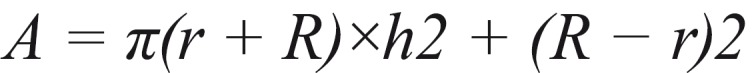
Formula.

In order to achieve similar bonding areas in all the three tests, the bonded root surface for the fiber posts in the two previous tests was calculated with the incomplete cone formula as follows (19,20), (Fig. 1):

By calculating the diameter of the post hole, which was 1.1 mm in the apical end and 1.2 mm in the cervical portions of the root slices, the “r” and “R” were 0.55 and 0.6 mm, respectively, with a height of 1.5 mm (20). Therefore, the diameter of the cylinder of the shear test was 3.2 mm by calculating the bonded area of the circle (πr2).

-Shear bond strength test

A total of 48 sound anterior teeth were used for the shear test. The tooth crowns were removed. The coronal aspect of cross-sections from the middle part of the roots were prepared and rinsed with NS and SHC in two equal groups as explained for the two previous tests. The plastic molds, measuring 3.2 mm in cross-section and 2 mm in length, were bonded in both Duo-Link and BisCem groups in root cross-sections according to manufacturers’ instructions. After 24 hours of storage in a water bath, the resin cylinders underwent a shearing force at a strain rate of 1 mm/min in a universal testing machine (Walter & Bai, K21046, Lohningen, Switzerland). Data was recorded and evaluated using SPSS statistical software *p*-value of <0.05 was set to be statistically significant.

For specimens of the three studied test methods, the fracture modes were evaluated under a light microscope at ×16 and classified as follows.

I. Cohesive fracture: fracture within the resin cement or dentin.

II. Adhesive fracture: fracture in the adhesive interface.

III. Mixed fracture: adhesive/cohesive fracture.

Statistical analyses

After bonding of the resin cements in the dimensions mentioned above for each test, each test was carried out and data was recorded. All the values were converted to MPa and evaluated by SPSS. Data of all the three tests were evaluated by ANOVA, and post hoc Tukey tests were used for two-by-two comparison of the groups. Weibull analysis was used to compare the three tests under study.

## Results

At first, Kolmogorov-Smirnov test was used to evaluate normal distribution of data, which showed *P*-values of 0.781, 0.284 and 1 for PS, MPL and S tests, respectively. In addition, data was homogeneous. Subsequently, univariate analysis of variance was used for each of the tests in the relevant subgroups.  Table 2 presents the results of bond strength tests in each subgroup for each test. The results showed more similarity between the means of bond strengths between shear and push-out tests. Therefore, there were no significant differences in the bond strength values in any of the two resin cement subgroups and SHC and NS subgroups of these two tests. However, the bond strengths of these two tests were significantly different from those of the modified pull-out test under all the conditions of the study. On the other hand, in all the three tests under study, use of sodium hypochlorite resulted in a mild decrease in bond strength means, which was noticeable but not statistically significant.

**Table 2:**
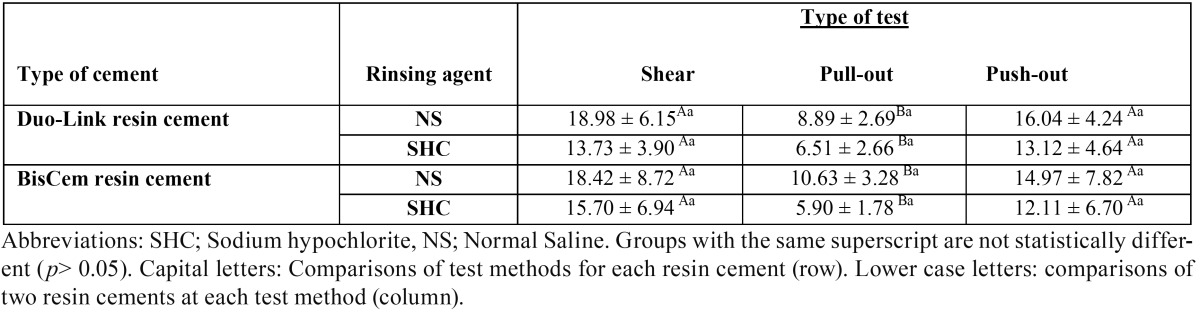
Bond strength (MPa) means ± standard deviation for Push-out, pull-out, and Shear tests for two studied resin cements and two rinsing protocols.

Comparison of bond strength means in corresponding groups with the use of different resin cements did not reveal any significant differences between etch-and-rinse and self-etch resin cements with the three tests (*P*>0.05).

**Figure 2:**
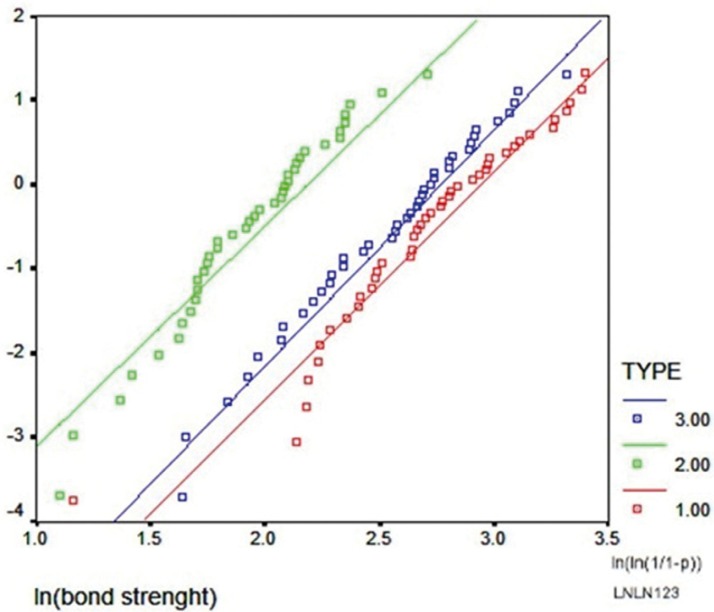
Weibull plot showing the results of Weibull modulus combined with the characteristic strength for three studied bond strength tests. (Red: Push-out, Green: Pull-out, Blue: Shear).

Weibull analysis and Weibull plot were used to compare the three push-out, modified pull-out and shear tests; the results are presented ( Table 3, Fig. 2). The characteristic strength values in the t Table 3 are as follows: push-out, 18.92 MPa, 2.92; modified pull-out, 8.89 MPa, 2.63; and shear, 15.80 MPa, 2.80. Distribution of Weibull coefficient (m) and ∂ᵒ values in the  Table 3, which indicates the characteristic strength in the three tests, showed a higher similarity between the two push-out and shear tests. However, considering the values and the plot obtained, all the three tests under study had relatively equal reliability coefficients.

**Table 3:**
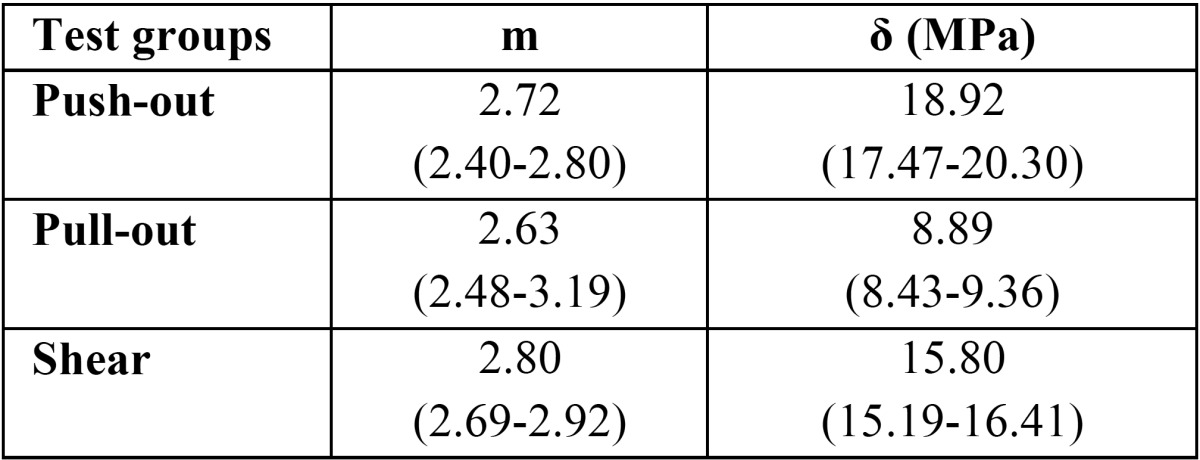
Weibull parameters (95% confidence intervals in parentheses), m –Weibull modulus, Ϭᵒ characteristic strength for Push-out, pull-out, and Shear.

Distribution of different failure modes in study groups are presented in  Table 4. There were not significant differences between three test methods regarding fracture mode.

**Table 4:**
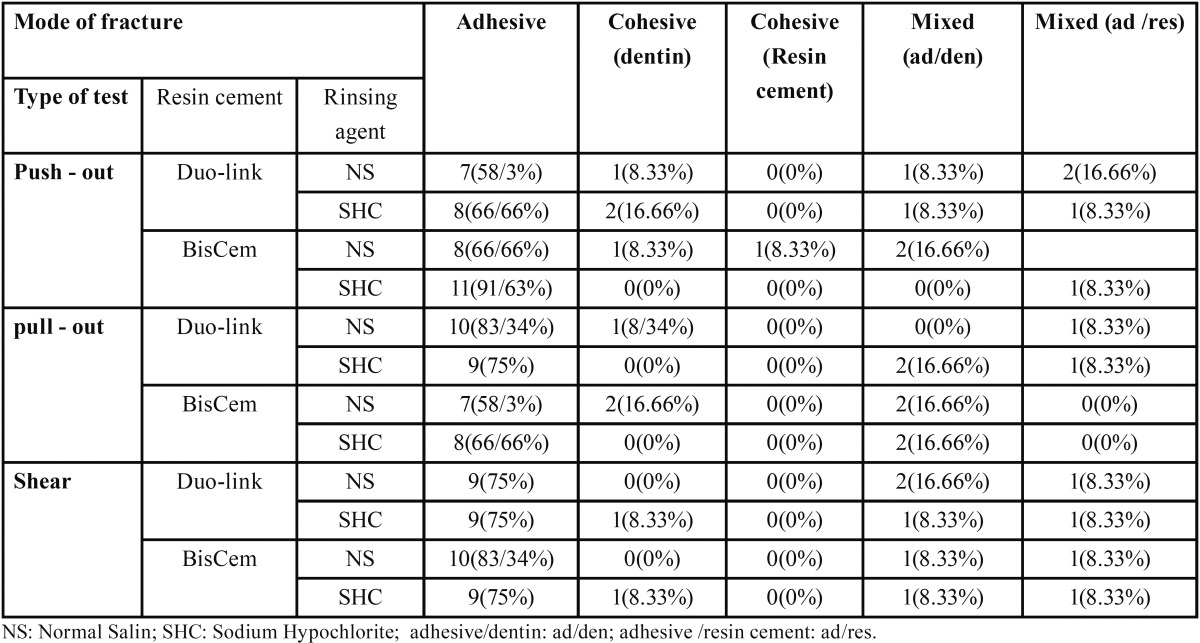
Distribution of different failure modes in study groups.

## Discussion

When the structure of a restoration is prepared based on the bond of the esthetic post with resin bonding cement and tooth dentin, its clinical success is especially dependent on the quality and durability of the bond between the tooth structure and the post. Therefore, it is necessary for the test method used for evaluating bond strength to be designed in a manner to yield correct and reliable data in relation to the behavior of the technique or the materials used. Specifically, an increase in the conformity of this behavior in the laboratory test with clinical behavior will result in the better use and application of the results in the clinic (19,21).

The bond between esthetic posts and tooth root is a complex one, which has been evaluated by various techniques in recent years. These techniques include a number of tests, of which the push-out, modified push-out, pull-out, modified pull-out, and microtensile bond strength tests with simple samples or in the form of an hourglass are the most commonly used ones and have been evaluated in various studies (7,19-21). In addition, evaluation of the bond strength of resin cement to root dentin by shear test has been introduced in some studies (22-24). On the other hand, bond strength tests are significantly under the influence of the geometric shape of the sample, the area of the bonded surface, the method of force application and also the type of the bonding agent and the resin cement (19,20).

Simpler tests such as shear and tensile tests have long been used routinely; however, they have some disadvantages. During the two past decades microtensile tests have been used to overcome some of these disadvantages (1,3,19,20). Microtensile tests, too, have disadvantages, especially when the samples have been prepared in the shape of an hourglass and consist of the fiber post on one hand and root dentin on the other; these disadvantages include premature failures before the test is carried out. However, at present in cases in which the aim of the study is the evaluation of the bond strength of root dentin, it is the most predictable test (19).

Pull-out test is considered the best test in relation to distribution of stress. In this context, this test can be used to more properly report the bond strength of fiber post to root dentin. However, a large number of fiber posts should be used for this test, which increases the cost of the study and decreases its popularity (7,19). In the push-out test, the force is applied parallel to the bonding interface, which results in the application of a shear stress and provides a better estimate of the bond strength (19).

In this context, a change in the geometric shape of the samples and the force application technique yields different bond strength values in the crown and root in different tests. Such differences in different experimental studies result in inability to compare the results of different studies with each other, which might sometimes even be contradictory (3,19).

In this study three bond strength tests – push-out, modified pull-out and shear tests – were used to evaluate the bond strength of fiber posts to root dentin by comparing two resin cements and two canal irrigation techniques. In other words, the researchers made an attempt to answer the research question whether the type of the test in terms of the cement type or preparation technique of the root has an effect on bond strength of resin cements to root dentin or not. The null hypothesis of the study stated that there are no differences in the mean bond strength values with the application of identical resin cement in identical canal irrigation protocol with the use of different tests. In the present study, in fact two factors of cement type and irrigation solution type were selected as the variables to control the final results of the three tests under study. Based on the null hypothesis, the results of bond strength test, which are somehow applicable to how the stresses are distributed, are not under the influence of the geometric form of the sample or how the forces are applied (pull, push, shear) when the cross-section of the bonded surface is the same. Based on the results of the study, it is possible to accept this hypothesis to some extent because it was acceptable for only shear and push-out tests.

The three test types selected are in fact among the most commonly used tests to evaluate bond strength of resin cement to root dentin in the presence of a post or without it in recent years, of which the shear test was carried out by bonding the resin cement to root dentin surface without using a post. Although the numeric values of each test in relation to its technique were different from those in other tests, clearly the effects of canal irrigation protocol and the type of the resin cement used, as main variables, showed similar variations in the tests.

In the present study, two resin cement types and two irrigation techniques (use of normal saline and sodium hypochlorite solution) were evaluated in all the three tests. Some previous studies have shown the effect of sodium hypochlorite as an agent reducing the bond strength (25-28). Based on some previous studies, use of this solution decreases the bond strength between composite resin and dentin (8,15,27,28). It has been reported that one of the reasons for this outcome is the residues and by-products of sodium hypochlorite, which have a negative effect on polymerization of adhesive systems. Use of sodium hypochlorite on dentin surfaces results in biologic oxidation, and the free radicals remaining as a result of oxidative effect of sodium hypochlorite on vinyl free radicals – which are produced as a result of light activation of the adhesive and are involved in its distribution and propagation – compete and lead to incomplete polymerization and premature termination of the polymer chain (27). In the present study, this factor was used to control the three tests. The authors intended to find out whether the effect of a variable, such as irrigation with sodium hypochlorite, would be the same in all the tests or not. According to the outcomes of the present study, the decreasing effect of this material was the same in all the tests assessed. However, its effect was not statistically significant, which might be attributed to the number of samples in each group. It appears the effect of sodium hypochlorite on bond strength might have become significant as a decreasing agent with an increase in the number of samples.

In addition, in this study two resin cements were compared. One of the cements, Duo-Link, needed preconditioning consisting of etching and use of an bonding agent before being bonded to dentin and the other, BisCem, was a self-etch self-adhesive cement and was able to bond to root dentin without any preconditioning (29). This factor, too, was somehow used as a control variable in the three tests so that comparison of data between these tests would provide better information about the effect of each test type. According to the outcomes, comparison of the two cement types with ANOVA showed no differences in bond strength between the cements in each test. However, there were differences in the means of bond strengths of the two resin cements in the three tests, with a significant difference between modified pull-out and the two other tests. In addition, the results of bond strengths of the two rein cements were similar in corresponding groups in terms of the effect of sodium hypochlorite. Some previous studies have shown that the type of the resin cement has a significant effect on the bond strength of fiber post to root dentin (29,30). In one study Rely-X Unicem self-adhesive cement exhibited a higher push-out bond strength compared to Rely-X ARC cement. Similarly, Ebert evaluated the bond strength of resin cements to root dentin using the pull-out test and reported that the effect of material and the cement type were significant on bond strength (7). In contrast, Calixto *et al.* reported that conventional resin cements were superior to the adhesive cements in relation to the bond strength (30).

In this *in vitro* study, Weibull test was used to compare the three tests. The results showed that the geometric shape of the sample and force application technique were effective on bond strength values. Therefore, from this point of view the null hypothesis of the study cannot be accepted. Based on cumulative results of data and their comparison with Weibull test, minor differences were observed between the tests because the minimum and maximum values did not overlap at 95% confidence interval. In this context, considering the gradient of the graph and the coefficient “m”, there was higher similarity between push-out and shear tests, which might be attributed to how the force is applied in these two tests, in which both compressive forces result in shearing. This similarity, especially in the present study in which the bonding area was similar in all the three tests, is of significance. In the study, based on the results of Weibull test and comparison of the mean bond strength values at 95% CI, although there were more similarities between shear and push-out tests compared to modified pull-out test, absence of overlapping of lower bound and upper bound values somehow reflect the differences between the three tests. In the shear and push-out tests, although the values 16.41 and 17.47 are close to each other, lack of overlapping is of significance and concern, necessitating further extensive evaluations with greater sample sizes.

Root cross-sections measuring 1.5 mm in length were used for the purpose of push-out and pull-out tests. All the canals were enlarged to the same extent using special drills. Based on some reports, smaller sizes of the cross-sections result in better distribution of force at the bonding interface; in this context, some recent studies, have used micro-sized cross-sections for these tests (3,24,31). In the study, the sizes were selected to create similarity in the bonding areas in all studied tests. In addition, in the modified pull-out test the samples were expected to be prepared and sectioned in a manner to leave a few millimeters of the post out of the root canal so that it could undergo a pulling action by the triscrew chuck, which was more practical and reproducible for the researchers.

Based on the results of the present study, shear test yielded results more similar to those of the push-out test, which were confirmed by the coefficient “m” and its gradient in the Weibull test ( Table 3, Fig. 2). The shear test is very easy to carry out and repeat and is more inexpensive than the push-out test. One of the most important advantages of this test is the fact that it does not need root canal treatment and use of a fiber post, which decreases the costs to a great extent. In addition, after bonding of the samples in this test only one bond interface will exist, which will undergo a shearing force and therefore it is more preferable to other tests.

In the present study, in order to perform the shear test, resin cements with bonding areas similar to the push-out and pull-out tests were bonded to dentin surfaces, which had been prepared from the middle regions of the roots. Bonding to this part of the root might be different from bonding within the root canal under the root canal treatment conditions, which is a limitation of the present study in relation to carrying out the shear test. Moreover, regarding c-factor, the shear test would not be able to mimic the canal conditions for bonding; the subject should be investigated in future studies.

Of course, further studies are necessary to compare different sections of root dentin and bond strength of resin materials by evaluating different variables, such as cement type, the type of the irrigation solution, sealer type and the effect of sealer residues and other variables during root canal treatment.

Finally, the researchers believe that any reliable test which measures the bond strength between the resin cement and root dentin should be easily reproducible and should also reveal the effect, albeit minor, of variables. By evaluation of the values obtained in this study and previous studies with different tests it can be concluded that comparison of mean numeric values in different studies and tests does not necessarily yield correct and reliable information. What is important and should be taken into account are changes in the means of bond strength values by evaluating the extent of increasing or decreasing gradient of the effect of each variable on bond strength.
